# Primary vs. Rescue Medium Vessel Occlusions: Comparative Clinical Outcomes in Patients with Acute Ischemic Stroke

**DOI:** 10.3390/jcm14228008

**Published:** 2025-11-12

**Authors:** Gokhan Ozdemir, Alper Eren, Nazim Kizildag, Omer Lutfi Gundogdu, Ayse Nur Ersoy, Muslu Kazim Korez, Leyla Ozturk Sonmez, Gozde Ongun, Nursen Unal, Uygar Utku

**Affiliations:** 1 Department of Neurology, Center of Stroke, Selcuk University Medical Faculty, Konya 42250, Turkey; aysenurersoymd@gmail.com; 2 Department of Neurology, Stroke Center, Ataturk University, Erzurum 25030, Turkey; alpereren25@gmail.com (A.E.); nazodiagnostic@hotmail.com (N.K.); 3 Department of Neurology, Medical Faculty, Recep Tayyip University, Rize 53020, Turkey; omerlutfigundogdu@gmail.com; 4 Department of Biostatistics, Selcuk University Medical Faculty, Konya 42250, Turkey; mkkorez@gmail.com; 5 Department of Neurology, Stroke Centre, Konya City Hospital, University of Health Sciences, Konya 42020, Turkey; ozturkleyla@gmail.com; 6 Department of Neurology, University of Istinye, Istanbul 34396, Turkey; gongun68@hotmail.com; 7 Department of Neurology, Stroke Centre, Ankara City Hospital, University of Health Sciences, Ankara 06170, Turkey; dr_nusiaslan@hotmail.com; 8 Kahramanmaras Stroke Centre, Kahramanmaras 46000, Turkey; utkuzar@gmail.com

**Keywords:** medium vessel occlusion (MeVO), rescue thrombectomy, primary MeVO, endovascular therapy (EVT), mechanical thrombectomy, ischemic stroke

## Abstract

**Background:** Medium vessel occlusions (MeVOs) are an increasingly recognized but heterogeneous target for endovascular therapy (EVT). This study aims to compare primary MeVO, rescue MeVO, and large vessel occlusion (LVO) thrombectomy cases to identify which MeVO subtypes derive a meaningful benefit from EVT under appropriate safety conditions. **Methods:** We retrospectively analyzed a multicenter registry of patients undergoing EVT for acute ischemic stroke. MeVO was defined as the occlusion of the A1-A3, M2-M3, P1-P3, fetal PCA, or PICA segments and classified as primary or rescue. Clinical outcomes were assessed by NIHSS score at baseline, discharge, and 90 days; functional outcome by the modified Rankin scale (mRS); and reperfusion by modified thrombolysis in cerebral infarction (mTICI). Safety endpoints included intracranial hemorrhage and mortality. **Results:** Among 603 EVT patients, 202 (33.5%) had MeVO. Compared to LVO, MeVO patients were older and had more prior strokes but achieved similar reperfusion and safety outcomes. At 90 days, mRS distribution differed, with MeVO showing more mRS 2 and LVO more mRS 1, while higher-disability strata were comparable. Within MeVO, 119 (58.9%) were primary and 83 (41.1%) rescue occlusions. Rescue MeVO patients presented with higher baseline severity (NIHSS score of 19 vs. 18) and, despite similar reperfusion, experienced worse 90-day outcomes and higher mortality (21.7% vs. 0.8%). **Conclusions:** EVT for primary MeVO is feasible, effective, and safe, whereas rescue MeVO is associated with poor functional outcomes and markedly higher mortality. These findings highlight rescue MeVO as a distinct phenotype and support a selective approach prioritizing disabling syndromes, proximal/dominant branch occlusions, and IVT non-response.

## 1. Introduction

Acute ischemic stroke is typically classified as large vessel occlusion (LVO) and non-large vessel occlusion (non-LVO) stroke. The safety and efficacy of endovascular treatment (EVT) for LVO have been validated by numerous randomized trials, and current recommendations advocate for EVT in suitably selected patients [1]. In contrast, for medium vessel occlusions (MeVOs) typically involving the M2-M3 segments of the middle cerebral artery, A2-A3 of the anterior cerebral artery, and P2-P3 of the posterior cerebral artery, high-level evidence has until recently been lacking [2,3,4].

MeVOs constitute around 25% to 40% of ischemic strokes and, despite lower infarct areas, can result in significant disabilities [2]. Although MeVOs are distinct from LVOs by caliber and segmental anatomy, clinical severity can overlap particularly in rescue MeVO. A subset of rescue cases initially present with proximal LVO, and the admission neurological deficit (e.g., NIHSS) reflects this global LVO burden rather than an isolated distal occlusion. In addition, distal occlusions in eloquent branches (language, hand motor, visual pathways) can yield disabling syndromes despite smaller vessel size. Accordingly, when interpreting baseline severity and subsequent outcomes, it is important to recognize that rescue MeVO may display LVO-like clinical severity, even though the target lesion at the time of distal thrombectomy is a MeVO.

Medium vessel occlusions (MeVOs) differ from large vessel occlusions (LVOs) not only by vessel caliber and location but also by the underlying etiopathogenesis. LVOs commonly arise from proximal atherothrombosis or cardioembolism producing longer, red blood cell-rich thrombi that lodge in large conduits (e.g., ICA terminus, M1) and are modulated by robust collateral networks. By contrast, MeVOs typically involve smaller, branching segments (M2–M3, A2–A3, P2–P3), where bifurcation geometry and higher shear favor distal embolic impaction and, in a subset, in situ thrombosis over distal atheroma or endothelial injury. Resultant clots tend to be shorter, more organized, and relatively fibrin/platelet-predominant, characteristics that influence IV thrombolysis responsiveness, spontaneous recanalization, and device clot interaction during endovascular therapy [3,5].

Intravenous thrombolysis (IVT) continues to be the usual initial treatment for MeVO; however, reperfusion and functional results are limited in comparison to proximal LVO, especially in cases with clinically significant impairments [2,6]. Motivated by enhanced operator proficiency and the accessibility of low-profile stent retrievers and aspiration equipment designed for smaller, more convoluted arteries, numerous facilities have broadened EVT to encompass MeVO targets [7].

Nevertheless, the initial randomized trials now moderate this enthusiasm. In ESCAPE-MeVO, the addition of EVT to standard treatment did not enhance 90-day outcomes compared to medical management in MeVO within 12 h [8]. Likewise, the DISTAL and DISCOUNT trials indicated no functional advantage of endovascular therapy compared to optimal conventional treatment for distal/medium vessel occlusions at 90 days, with indications of increased mortality in certain analyses [9,10]. The data underscore the heterogeneity of MeVO (vessel size/location, NIHSS, collaterals, time to reperfusion) and imply that any potential advantage of EVT if it exists may be restricted to precisely delineated subgroups (e.g., proximal M2 with significant deficiencies or IVT failure) [2,7].

Given the absence of a universal MeVO taxonomy and the clinical reality that rescue MeVO reflects LVO-context distal occlusions, pooled “All-MeVO” summaries are inherently heterogeneous. Accordingly, outcomes are primarily reported separately for primary (de novo) MeVO and rescue MeVO. Because distal emboli may be present before EVT (concomitant with proximal LVO) or may arise during EVT, “rescue MeVO” is used as a context-defined category: distal occlusions recognized during or immediately after proximal thrombectomy, without inference about the timing or causality of embolization. In contrast, “primary (de novo) MeVO” denotes a distal target lesion at initial presentation that is treated without preceding proximal thrombectomy. This pragmatic taxonomy enables comparison between primary MeVO and LVO-context distal occlusions (rescue MeVO) while acknowledging the absence of a universally accepted MeVO classification.

In light of this growing context, we sought to assess the efficacy, safety, and practicality of mechanical thrombectomy for MeVOs in our practice, encompassing both primary and rescue purposes. Our aim is to provide comprehensive clinical and procedural data to help identify which MeVO subtypes may derive a net clinical benefit from endovascular therapy (EVT) while maintaining rigorous safety standards.

## 2. Methods

### 2.1. Study Design and Patients

We retrospectively analyzed a prospectively maintained registry of consecutive adults undergoing mechanical thrombectomy (MT) for acute ischemic stroke (2017–2024). Medium vessel occlusion (MeVO) was defined as the occlusion of A1–A3, M2–M3, P1–P3, fetal PCA, or PICA segments and classified as primary (de novo) or rescue (distal embolization during/after LVO therapy). Rescue MeVO was defined as a distal occlusion identified during or immediately after proximal EVT for an index LVO. This category intentionally captures LVO-context distal occlusions and does not assume an iatrogenic mechanism; the timing (pre-procedural vs. intra-procedural) could not be adjudicated consistently and was not used for classification. Primary MeVO denoted a de novo distal target without preceding proximal thrombectomy. Any pooled “All-MeVO” estimates are presented for completeness but considered heterogeneous due to the inclusion of LVO-context distal occlusions (rescue). EVT for primary MeVO was considered in the presence of disabling deficits (even with lower NIHSS score), eloquent territory involvement, perfusion mismatch, long/organized thrombus, or non-response/early worsening after IVT. Two independent interventional neurologists adjudicated occlusion type.

### 2.2. Procedure

All patients were managed under standardized acute stroke protocols (non-contrast CT with CTA ± perfusion; IV alteplase if eligible). MT was performed with low-profile stent retrievers, under conscious sedation or general anesthesia or without sedation at operator discretion. Adjunctive intra-arterial thrombolysis was permitted. Balloon-guide or aspiration catheters were not routinely used for MeVO. Rescue MeVO was identified through selective angiography after each LVO pass.

### 2.3. Devices and Sizing

Mechanical thrombectomy was performed with stent retrievers in both LVO and MeVO. We used Solitaire™ X (Medtronic, Dublin, Ireland), APERIO^®^ Hybrid/Hybrid17|21 (Acandis, Pforzheim, Germany), and CATCH/CATCH Mini/CATCHVIEW (Balt, Montmorency, France). Device choice and size were operator-determined by occlusion site and estimated vessel diameter. For MeVO, we preferentially selected smaller-caliber retrievers (e.g., Solitaire X 3 × 20 mm; APERIO 2.5–3.5 mm diameters in 16–40 mm lengths; CATCH Mini 3 × 15–20 mm), while larger diameters/lengths were used for LVO (e.g., Solitaire X 4–6 mm diameter with 20–40 mm working length; CATCHVIEW 4 × 20 mm and CATCHVIEW MAXI 6 × 30 mm). Microcatheter compatibility followed IFU (APERIO Hybrid17 allows 0.0165–0.021″ ID; standard APERIO 0.021″ ID).

### 2.4. Outcomes

The primary endpoint was successful reperfusion (final mTICI 2b–3). Secondary outcomes included NIHSS score at baseline, discharge, and 90 days; functional outcome (mRS, favorable = 0–2); and safety outcomes (symptomatic ICH, any ICH, early clinical worsening, and mortality).

### 2.5. Statistics

Analyses were performed in R v4.2.1. Continuous variables were summarized as the mean ± SD or median [IQR], categorical variables as *n* (%). Group comparisons used χ^2^, Fisher’s exact, t-test, or Mann–Whitney U as appropriate. Logistic regression was applied to explore factors associated with MeVO and with primary versus rescue phenotypes. Significance was set at *p* < 0.05.

### 2.6. Ethics Statement

This retrospective multicenter registry study was approved by the Selcuk University, Medical Faculty (Approval No: E-70632468-050.04-666549). In accordance with institutional policy, the requirement for individual informed consent was not required, because all analyses used identified data collected during routine clinical care and posed minimal risk to participants. This study adhered to the principles of the Declaration of Helsinki.

## 3. Results

**Figure 1:**
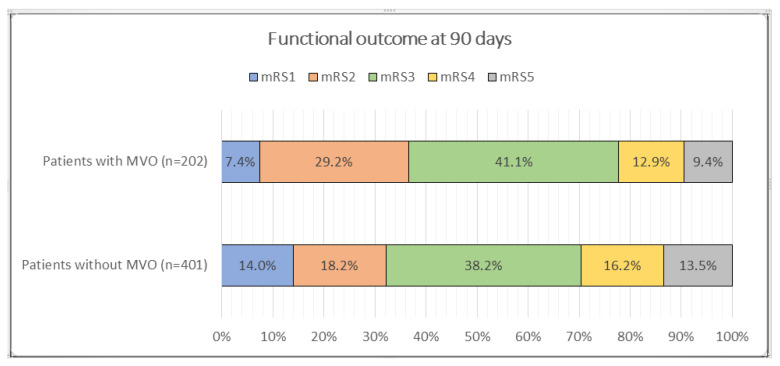
Comparison of functional outcome by mRS score in 3rd month between patients with and without MeVOs.

Among 603 thrombectomy patients, 401 (66.5%) had large vessel occlusion (LVO), and 202 (33.5%) had medium vessel occlusion (MeVO) (Table 1). MeVO patients were older than LVO patients (71.26 ± 14.04 vs. 67.28 ± 15.17 years, *p* = 0.002). Sex distribution was similar (49.5% vs. 48.1% male, *p* = 0.750). Vascular risk profiles did not differ significantly between groups for hypertension (70.3% vs. 65.3%, *p* = 0.221), diabetes mellitus (16.3% vs. 21.4%, *p* = 0.137), dyslipidemia (24.8% vs. 22.2%, *p* = 0.481), atrial fibrillation (27.7% vs. 25.7%, *p* = 0.592), smoking (16.3% vs. 20.0%, *p* = 0.283), or heart valve replacement (1.5% vs. 2.7%, *p* = 0.404). A prior stroke was more common in MeVO (23.3% vs. 14.7%, *p* = 0.009). The use of intravenous tPA was comparable (32.7% vs. 34.2%, *p* = 0.715). The median puncture time was slightly shorter in MeVO (190 (120–274) vs. 210 (140–300) minutes) without statistical significance (*p* = 0.080). The puncture-to-recanalization time was similar (40 (30–55) vs. 38 (29–50) minutes, *p* = 0.403). Angiographic reperfusion did not differ between groups (*p* = 0.589): mTICI 2b–3 was achieved in 61.4% (MeVO) vs. 64.3% (LVO) and complete reperfusion (mTICI 3) in 32.7% vs. 31.4%, respectively. Baseline stroke severity was comparable (admission NIHSS score of 18 (17–21) vs. 18 (15–21), *p* = 0.420). The NIHSS score at 3 months did not differ (10 (4–13) vs. 10 (8–14), *p* = 0.221) (Figure 1). The 90-day mRS distribution differed significantly (*p* = 0.004). Pairwise comparisons indicated higher mRS 1 in LVO (14.0%) and higher mRS 2 in MeVO (29.2%), while the proportions at mRS 3–5 were broadly similar (Table 1). The rates of intracranial hematoma were nearly identical (17.3% MeVO vs. 17.2% LVO, *p* = 0.971). Mortality was numerically lower in MeVO (9.4% vs. 13.5%) but not statistically significant (*p* = 0.190). Overall, compared with LVO, MeVO cases in this cohort were older and more likely to have a history of stroke, yet they demonstrated similar reperfusion success, procedure times, neurological severity, and safety, with a modest shift in functional outcomes favoring mRS 2 in MeVO and mRS 1 in LVO.

Among 520 patients included in the LVO–primary MeVO comparison, baseline demographics and vascular risk factors were broadly similar except for a slightly higher mean age in primary MeVO (70.8 ± 14.2 vs. 67.3 ± 15.2 years, *p* = 0.002). Procedure times and reperfusion grades (mTICI 2b–3 ≈ 64%) were comparable. Despite similar technical outcomes, primary MeVO achieved a higher rate of functional independence at 90 days (mRS 0–2: 47.9% vs. 32.2%, *p* = 0.003) and lower mortality (0.8% vs. 13.5%, *p* < 0.001). These findings suggest that, under expert-driven selection (eloquent territory, functionally disabling deficits, or perfusion mismatch), primary MeVO thrombectomy yields outcomes comparable to or better than LVO despite the smaller vessel caliber (Table 2).

### 3.1. Primary Versus Rescue MeVO

Among 202 MeVO cases, 119 (58.9%) were primary and 83 (41.1%) were rescue occlusions (Table 3). Age and sex were comparable between groups (70.76 ± 14.18 vs. 71.98 ± 13.88 years, *p* = 0.545; 49.6% vs. 49.4% male, *p* = 0.980). Vascular risk profiles did not differ significantly for hypertension (72.3% vs. 67.5%, *p* = 0.563), diabetes mellitus (17.6% vs. 14.5%, *p* = 0.682), dyslipidemia (22.7% vs. 27.7%, *p* = 0.517), atrial fibrillation (26.1% vs. 30.1%, *p* = 0.634), smoking (15.1% vs. 18.1%, *p* = 0.716), heart valve replacement (2.5% vs. 0%, *p* = 0.270), or prior stroke (21.8% vs. 25.3%, *p* = 0.688). The use of IV tPA was similar (38/119, 31.9% vs. 28/83, 33.7%; *p* = 0.788). The median puncture time showed a non-significant trend toward a delay in rescue MeVO (210 (140–300) vs. 180 (120–260) minutes, *p* = 0.150). Puncture-to-recanalization times were comparable (38 (29–60) vs. 40 (30–55) minutes, *p* = 0.854). Angiographic reperfusion rates did not differ overall (*p* = 0.178): no reperfusion in 9.6% vs. 3.4%, mTICI 2b–3 in 57.8% vs. 63.9%, and mTICI 3 in 32.5% vs. 32.8% (rescue vs. primary, respectively) (Table 3).

### 3.2. Neurological Severity, Functional Outcomes, and Safety

**Figure 2:**
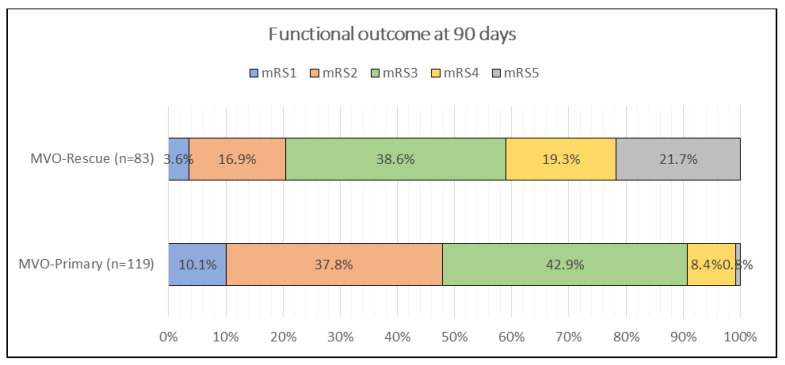
Comparison of functional outcome by mRS score in 3rd month between patients with primary and rescue MeVOs.

Admission stroke severity was higher in rescue MeVO (NIHSS score of 19 (17–25) vs. 18 (17–19), *p* = 0.0016). At 3 months, the NIHSS score remained worse in rescue cases (13 (8–15) vs. 9.5 (4–12), *p* < 0.001). The 90-day mRS distribution differed significantly (*p* < 0.001); post hoc contrasts (see superscripts in Table 2) showed that mRS 2 was more frequent after primary MeVO (37.8% vs. 16.9%), whereas mRS 4 (19.3% vs. 8.4%) and mRS 5 (21.7% vs. 0.8%) were more common after rescue MeVO (Figure 2). Intracranial hematoma rates were similar (19.3% vs. 16.0%, *p* = 0.672). Mortality was substantially higher in rescue MeVO (21.7% vs. 0.8%, *p* < 0.001) (Table 4).

### 3.3. Interpretation

In comparison to main MeVO, rescue MeVO exhibited increased initial severity and, despite analogous procedure durations and reperfusion grades, was linked to poorer 90-day neurological and functional outcomes, as well as significantly elevated mortality rates, whereas hemorrhagic complications were identical.

## 4. Discussion

This multicenter registry offers an extensive, real-world depiction of endovascular therapy (EVT) utilized for medium vessel occlusion (MeVO) in conjunction with large vessel occlusion (LVO) during a seven-year duration. A multitude of messages arise. First, MeVO constituted roughly one third of all thrombectomy cases, indicating that distal targets are already a substantial part of daily practice. Compared with LVO patients, MeVO patients were older and had a higher prevalence of prior stroke, yet they achieved similar angiographic reperfusion (mTICI 2b–3 and complete mTICI 3), comparable puncture-to-recanalization times, and near-identical rates of intracranial hematoma and mortality. Functional outcomes at 90 days demonstrated little distributional difference. LVO exhibited a higher prevalence of mRS 1, whereas MeVO showed a greater occurrence of mRS 2 without differentiation in the more severely impaired categories. Collectively, these data endorse the practical viability and overall safety of EVT in MeVO within modern workflows while also warning that the extent of population-level functional advantage is probably less significant and more variable than that for proximal LVO.

A direct comparison between primary MeVO and LVO in our cohort offers further insight into the efficacy of distal occlusion thrombectomy under expert-driven patient selection. Despite comparable reperfusion rates, primary MeVO cases achieved a markedly higher rate of functional independence (47.9% vs. 32.2%) and significantly lower mortality (0.8% vs. 13.5%). These findings indicate that when appropriately selected, such as for patients with eloquent territory infarctions, disabling symptoms despite a low NIHSS score, or imaging evidence of salvageable penumbra, primary MeVO thrombectomy can yield outcomes at least equivalent to, or even better than, those of LVO. This supports the concept that clinical and imaging context, rather than vessel caliber alone, should guide endovascular decision-making in MeVO.

Our results complement and extend the observations of the DISTAL and ESCAPE-MeVO randomized trials, which primarily enrolled populations corresponding to primary MeVO as defined in our study. Both trials highlighted the heterogeneity of MeVO presentations and the importance of individualized patient selection. In this context, our high recanalization rate (~90%) and favorable outcomes reinforce the notion that, in centers with advanced neurointerventional expertise and well-structured stroke workflows, carefully selected primary MeVO patients may derive meaningful benefit from mechanical thrombectomy. Conversely, the poorer outcomes observed in rescue MeVO emphasize procedural complexity and the confounding influence of initial LVO-related severity, underscoring the need for prospective, mechanism-specific trials to refine patient selection and procedural strategies.

A plausible rationale for thrombectomy in MeVO extends beyond vessel caliber and includes clot biology, branching/bifurcation anatomy, and clinical–penumbral dynamics. Distal clots are often short yet more organized and fibrin/platelet-predominant, features associated with reduced responsiveness to IV thrombolysis and lower rates of spontaneous recanalization. In addition, bifurcation geometry and small-caliber, high-friction segments can promote distal impaction and increase the risk of fragmentation after IVT. Clinically, MeVOs frequently involve eloquent territories (language, hand motor cortex, visual pathways); even with a small core, they may cause disabling deficits and demonstrate perfusion–diffusion mismatch indicative of salvageable penumbra. Contemporary microcatheters and appropriately sized stent retrievers and/or aspiration techniques can mitigate these biomechanical disadvantages with high technical success and an acceptable safety profile. Accordingly, thrombectomy in MeVO is a time-sensitive and rational option in patients with disabling symptoms, IVT non-response or early neurological worsening, perfusion imaging mismatch, long clots/low permeability, or distal embolization after proximal LVO thrombectomy (rescue MeVO).

Conceptually, rescue MeVO represents a composite clinical context of LVO-associated distal occlusion rather than a single etiologic pathway. Distal embolization may pre-exist at presentation or emerge during EVT, and our data do not permit case-by-case timing adjudication. We therefore avoid causal statements about iatrogenic migration and interpret the inferior outcomes of rescue MeVO as reflecting the clinical context and case complexity of LVO-associated distal occlusions, rather than an intrinsic property of the distal lesion alone.

The most therapeutically significant observation is the distinction between main and rescue MeVO. Rescue MeVO, characterized by distal embolization or clot fragmentation observed during or following proximal LVO therapy, exhibited greater baseline severity and resulted in poorer neurological and functional outcomes, accompanied by significantly elevated mortality rates (21.7% vs. 0.8%), despite comparable reperfusion grades to primary MeVO. This pattern strongly suggests that rescue MeVO is not simply an alternative target location but a unique pathophysiological phenotype. Multiple convergent mechanisms may elucidate the gradient: (i) an increased initial ischemic burden from the primary major vessel blockage prior to distal embolization; (ii) embolic debris dispersing into several distal branches, augmenting the final infarct volume even when a singular dominant occlusion is recanalized; (iii) microvascular no-reflow and microemboli that impair tissue-level reperfusion without affecting mTICI; and (iv) time penalties associated with successive treatments, which may not be reflected in median puncture-to-recanalization timings but remain biologically significant [10]. The divergence between comparable mTICI scores and poorer clinical outcomes in rescue situations MeVO emphasizes the inadequacies of traditional angiographic surrogates in limited regions and reinforces the necessity for metrics that more accurately represent parenchymal salvage (e.g., tICI 2c-3, first-pass effect, distal embolus counts) when assessing MeVO strategies.

Despite growing interest, there is no universally accepted classification system for medium vessel occlusions (MeVOs); existing schemes vary by anatomic segment, clot biology, and treatment context. While M2 is often categorized as “medium-vessel,” some classifications consider it a large-artery segment; moreover, M2 is frequently overrepresented in prior cohorts, potentially biasing outcomes toward larger-vessel strokes and limiting generalizability to more distal phenotypes (M3–M4, A2–A3, P2–P3). In our study, MeVO was defined as the occlusion of A1–A3, M2–M3, P1–P3, fetal-type PCA, or PICA segments. Another source of heterogeneity is the inconsistent distinction between primary MeVOs and secondary (rescue) MeVOs, which differ substantially in ischemic burden and procedural course; pooling them may obscure true effects. Furthermore, while MeVO-eTICI has been proposed for small-territory reperfusion, it lacks universal validation, hindering cross-trial comparability. Finally, the incomplete reporting of IV thrombolysis as a key modifier in MeVO management remains a recurrent limitation. Future trials should therefore (i) prespecify vascular segment strata, (ii) distinguish primary from rescue MeVOs, (iii) adopt validated reperfusion metrics, and (iv) ensure the complete reporting of co-interventions. Such refinements will improve interpretability and yield more clinically relevant estimates of EVT benefit in MeVO stroke.

Our findings align with recent randomized trials, which, despite high technical success and acceptable safety, did not demonstrate a consistent 90-day functional benefit of EVT over the best medical therapy in broadly inclusive MeVO populations [7,8,9]. Although our study lacked a medical therapy comparator, the neutral differences between MeVO and LVO, and particularly the divergence between primary and rescue MeVO, support the view that EVT efficacy in MeVO is heterogeneous and confined to select phenotypes. Thus, MeVO should not be treated as a single entity: a proximal/dominant M2 occlusion with disabling deficits differs fundamentally from a minor distal M3/A3/P3 occlusion with a low NIHSS score and high likelihood of IVT response.

The procedural details from this cohort provide more clarity. MeVO interventions utilized low-profile stent retrievers and selective intra-arterial alteplase as necessary; aspiration catheters, intermediate catheters, and balloon-guide catheters (BGCs) were excluded for MeVO targets. This technique is understandable in tortuous, small-caliber vessels where trackability and perforator safety are priorities. However, the adverse outcomes concentrated in rescue MeVO prompt reflection on the upstream LVO strategy that precedes many rescue cases. Proximal thrombectomy, with proximal flow control and aspiration support, has been linked to decreased distal embolization and enhanced first-pass efficacy in various observational datasets. While our dataset was not intended to evaluate hardware causality, the juxtaposition of (a) comparable mTICI scores and (b) significantly poorer outcomes in rescue MeVO strongly advocates for the implementation of embolization-minimizing strategies during LVO therapy. This includes, for instance, routine BGC when anatomically permissible, prudent pass counts with timely technique alterations upon suboptimal engagement, careful clot management to prevent fragmentation, and systematic selective oblique angiography after each pass to promptly identify and address clinically significant distal emboli. Such process improvements could reduce the incidence and severity of the rescue MeVO phenotype without requiring universal escalation at the distal target itself [11].

Our findings should be interpreted with the understanding that rescue MeVO frequently exhibits LVO-like clinical severity. This pattern is biologically and clinically plausible for two reasons: (i) a proportion of rescue cases presented with LVO at baseline, so the admission NIHSS score largely captures the initial proximal occlusion deficit, and (ii) eloquent distal territories can produce profound disability even when the index vessel is small. For this reason, we avoided attributing the admission NIHSS score solely to MeVO in the rescue cohort, prioritized 90-day functional outcomes for prognostic comparisons, and reported baseline severity separately for primary MeVO where appropriate. These considerations help reconcile the observed discordance between similar reperfusion grades and worse outcomes in rescue MeVO and underscore the need for workflow strategies that minimize distal embolization and expedite earlier definitive therapy in eligible primary MeVO presentations.

Patient selection is identified as another crucial factor. The observed overall safety indicates that MeVO EVT may be deemed appropriate when a distinct immediate neurological advantage is likely; nonetheless, the current findings do not endorse the routine treatment of unselected MeVOs. A pragmatic selection framework consistent with our findings would emphasize the following: (i) the alleviation of clinical syndromes resulting from the occlusion (e.g., global aphasia, dense hemiparesis, hemianopia/neglect in the dominant hemisphere), (ii) proximal or dominant branch occlusions (e.g., proximal/dominant M2) exhibiting favorable imaging (small core, salvageable tissue), and (iii) the absence of response to IV thrombolysis when rapid reperfusion is not attained. In contrast, for distal, low-NIHSS-score occlusions in non-eloquent regions or with evident early improvement, conservative therapy accompanied by rigorous surveillance may be more advantageous. Prospectively implementing this strategy could focus EVT utilization in enriched subgroups more likely to benefit from it while mitigating harm and resource expenditure in lower-yield situations [12]. The M3 and A2 segments provide blood to the supplementary and primary motor areas; obstruction in these regions may result in aphasia and hemiparesis, as noted in our sample. These instances demonstrate the necessity of a selected, phenotype-driven strategy: distal occlusions in non-eloquent areas may require conservative observation, while proximal or eloquent-branch MeVOs resulting in debilitating symptoms should be prioritized for EVT evaluation.

Several additional observations are noteworthy. The modest shift in 90-day mRS with MeVO showing more mRS 2 and LVO more mRS 1 likely reflects territory size and symptom profile: small cortical infarcts often leave domain-specific deficits (aphasia, neglect, fine motor loss) that result in mRS 2 rather than full independence. This supports complementing global mRS with domain-level endpoints in MeVO research. Older age and higher prior stroke prevalence in MeVO may also explain functional plateaus despite adequate reperfusion, underscoring the role of pre-morbid reserve. Furthermore, improvements in devices and operator experience between 2017 and 2023 likely contributed to the observed safety and reperfusion rates. Critically, outcomes are further influenced by the lack of distinction between primary and rescue MeVOs, as rescue cases carry greater ischemic burden and embolic load and longer procedures. Distal embolism may impair collaterals and accelerate penumbral loss, causing additional ischemia. Without this separation, the functional results may appear attenuated and temporal progress underestimated. Future studies should therefore explicitly stratify primary and rescue MeVOs.

In current endovascular practice, mechanical thrombectomy for medium vessel occlusions (MeVOs) is often regarded as technically more demanding due to the distal location of the clot and the increased procedural complexity. Moreover, medium-caliber arteries are inherently fragile, necessitating heightened vigilance for hemorrhagic complications, vascular dissection, and perforation. Nonetheless, increasing operator experience combined with advances in device technology has led to greater confidence in extending EVT to MeVO targets. Despite this, patients with MeVO have historically been underrepresented in pivotal thrombectomy trials, and robust data specifically addressing the safety and efficacy of thrombectomy in this context remain limited [13,14].

This research possesses limitations. This is a retrospective analysis of a prospectively held registry; hence it is susceptible to selection bias and unmeasured confounding variables. Vascular imaging paths prior to procedures differed; certain lesions were predominantly identified using emergent DSA instead of CTA/MRA, potentially impacting triage and temporal metrics. Our MeVO definition was intentionally broad (including A1, P1, PICA in addition to M2–M3/A2–A3/P2–P3), increasing biological and technical heterogeneity and potentially diluting effects in any single territory. Also, we did not capture multi-territory/multifocal infarction in our dataset; consequently, we could not evaluate its potential confounding effect on functional outcomes, and no causal inference regarding this factor can be made from our data. Because patient selection incorporated clinical disability and imaging-based treatability rather than NIHSS score alone, some degree of selection bias toward moderate-to-severe or “treatable” MeVO may persist; the results should be interpreted as practice-reflective and hypothesis-generating. Device choices reflected institutional practice (no aspiration/BGC for MeVO), limiting generalizability to centers that employ alternative strategies. The angiographic and clinical outcomes were not evaluated by a core laboratory; although sICH was defined a priori, the bleeding data at the table level recorded “hematoma” instead of adjudicated sICH, potentially leading to a misclassification of symptomatic occurrences. In conclusion, while we present strong univariate comparisons, this manuscript does not encompass adjusted modeling for all endpoints or territory-specific subgroup analyses (e.g., dominant vs. non-dominant M2, proximal vs. distal M2/M3, anterior vs. posterior MeVO), both of which are intended for future research.

Direct primary MeVO vs. LVO comparison clarifies that clinical prognosis reflects not only reperfusion but also context and severity. For rescue MeVO, our data show that achieving distal reperfusion does not necessarily offset the adverse clinical context: initial LVO-level severity, potential injury to eloquent distal territories, and procedural complexity likely contribute to the higher mortality observed. Identifying predisposing anatomical/procedural factors for LVO-context distal occlusions and determining when immediate distal revascularization changes outcomes are key priorities for prospective studies.

The inferior outcomes observed in rescue MeVO should not necessarily be taken as evidence against rescue therapy. Instead, they may reflect a higher-risk clinical context including initial LVO-level severity, eloquent distal involvement, and procedural complexity. A more definitive assessment of the incremental effect of rescue would require the prospective, protocolized capture of all distal migration events (treated and untreated) with predefined rescue criteria, to help mitigate confounding by indication.

The clinical implications are actionable. Endovascular treatment (EVT) for MeVO demonstrates safety and technological feasibility at proficient centers; nevertheless, the benefits are not consistent. Our data advocate for a selective, phenotype-driven strategy: to address debilitating, physically advantageous MeVOs (especially proximal/dominant M2) and exercise caution with distal, low-severity occlusions until there is a demonstrated risk to eloquent cortex. Equally important is preventing rescue MeVO during LVO thrombectomy by minimizing distal embolization and surveilling for new distal occlusions in real time. Looking forward, prospective studies should (i) validate selection rules that integrate clinical severity, eloquence, and perfusion core mismatch for small territories; (ii) test standardized hardware algorithms that incorporate proximal flow control where feasible; (iii) incorporate core lab imaging with distal embolus quantification and perfusion endpoints; and (iv) adopt domain-specific functional measures alongside mRS to better align angiographic success with patient-centered recovery. In the absence of such evidence, the existing data including the current analysis advocates for targeted rather than regular EVT for MeVO and increased vigilance to block the high-risk rescue MeVO route. Also, combining primary and rescue MeVO introduces population heterogeneity and LVO-context characteristics; thus, the pooled MeVO cohort does not represent a strictly “pure” de novo MeVO population. This registry inadequately documented instances of untreated distal migration during LVO thrombectomy; hence, a thorough comparison of treated and untreated rescue MeVO was unfeasible. Therefore, our study is unable to measure the additional advantage of rescue thrombectomy in cases of LVO-associated distal emboli. Nevertheless, the current research provides a systematic, practical paradigm that differentiates primary (de novo) from rescue (LVO-context) MeVO, thereby minimizing variability and elucidating unique pathophysiological and procedural settings within the MeVO spectrum. This classification establishes a foundation for more uniform comparisons such as our new primary MeVO versus LVO analysis and may guide future prospective studies, especially in enhancing patient selection, optimizing workflows, and designing endpoints in endovascular therapy research.

## 5. Conclusions

Mechanical thrombectomy for primary MeVOs is effective, safe, and feasible. Conversely, rescue MeVOs demonstrate inadequate clinical results despite similar procedure durations, reperfusion levels, and bleeding rates, accompanied by significantly elevated 90-day mortality. These findings advocate for prioritizing early definitive thrombectomy in suitable main MeVO cases and enhancing standardized rescue protocols; validation through prospective multicenter research is necessary.

## Figures and Tables

**Table 1:** The demographical characteristics, clinical presentations, and outcomes of patients with LVO and MeVOs.

Characteristics	Patients with LVO (*n* = 401)	Patients with MeVOs (*n* = 202)	*p*-Value
Demographical characteristics			
Age (years), mean ± SD (range)	67.28 ± 15.17 (17–105)	71.26 ± 14.04 (14–96)	0.002 ^1^
Gender (male), *n* (%)	193 (48.1)	100 (49.5)	0.750 ^2^
Medical history (presence)			
Hypertension, *n* (%)	262 (65.3)	142 (70.3)	0.221 ^2^
Diabetes mellitus, *n* (%)	86 (21.4)	33 (16.3)	0.137 ^2^
Dyslipidemia, *n* (%)	89 (22.2)	50 (24.8)	0.481 ^2^
Atrial fibrillation, *n* (%)	103 (25.7)	56 (27.7)	0.592 ^2^
Smoking, *n* (%)	80 (20)	33 (16.3)	0.283 ^2^
Heart valve replacement, *n* (%)	11 (2.7)	3 (1.5)	0.404 ^3^
A history of stroke, *n* (%)	59 (14.7)	47 (23.3)	0.009 ^2^
Intravenous tPA, *n* (%)	137 (34.2)	66 (32.7)	0.715 ^2^
Reperfusion, *n* (%)			0.589 ^2^
None	17 (4.2)	12 (5.9)	
mTICI 2b-3	258 (64.3)	124 (61.4)	
mTICI 3	126 (31.4)	66 (32.7)	
Puncture time, median (IQR)	210 (140–300)	190 (120–274)	0.079 ^5^
Puncture-to-recanalization time, median (IQR)	38 (29–50)	40 (30–55)	0.402 ^5^
Admission NIHSS score, median (IQR)	18 (15–21)	18 (17–21)	0.419 ^5^
NIHSS score in the 3rd month, median (IQR)	10 (8–14)	10 (4–13)	0.220 ^5^
mRS score in the 3rd month, *n* (%)			0.004 ^2^
mRS1	56 (14) ^a^	15 (7.4) ^b^	
mRS2	73 (18.2) ^a^	59 (29.2) ^b^	
mRS3	153 (38.2)	83 (41.1)	
mRS4	65 (16.2)	26 (12.9)	
mRS5	54 (13.5)	19 (9.4)	
Hematoma, *n* (%)	69 (17.2)	35 (17.3)	0.971 ^2^
Mortality, *n* (%)	54 (13.5)	19 (9.4)	0.190 ^4^

Data are presented as mean ± SD (ranges: min–max) or median with interquartile range (IQR) for numerical variables; categorical variables are described as number (*n*) and percentage (%). Different small superscript letters in each row show statistically significant differences between groups. ^1^ Independent sample *t*-test. ^2^ Pearson chi-square test. ^3^ Fisher’s exact test. ^4^ Chi-square test with Yates continuity correction. ^5^ Mann–Whitney *U* test. SD: standard deviation; tPA: tissue plasminogen activator; mTICI 2b-3: modified thrombolysis in cerebral infarction; NIHSS: National Institutes of Health Stroke Scale; mRS: modified Rankin scale; IQR: interquartile range.

**Table 2:** Comparison of LVO and Primary MeVO thrombectomy cohorts.

	LVO (*n* = 401)	Primary MeVO (*n* = 119)	*p*
Age (years)	67.28 ± 15.17	70.76 ± 14.18	0.002
Male sex, *n* (%)	193 (48.1)	59 (49.6)	0.750
Hypertension, *n* (%)	262 (65.3)	86 (72.3)	0.221
Diabetes mellitus, *n* (%)	86 (21.4)	21 (17.6)	0.137
Atrial fibrillation, *n* (%)	103 (25.7)	31 (26.1)	0.592
Smoking, *n* (%)	80 (20.0)	18 (15.1)	0.283
Intravenous tPA, *n* (%)	137 (34.2)	38 (31.9)	0.715
Puncture time, min (IQR)	210 (140–300)	180 (120–260)	0.080
Puncture to recanalization, min (IQR)	38 (29–50)	40 (30–55)	0.403
mTICI 2b–3, *n* (%)	258 (64.3)	76 (63.9)	0.964
mTICI 3, *n* (%)	126 (31.4)	39 (32.8)	0.802
Admission NIHSS score (IQR)	18 (15–21)	18 (17–19)	0.420
3-month NIHSS score (IQR)	10 (8–14)	9.5 (4–12)	0.221
mRS 0–2, *n* (%)	129 (32.2)	57 (47.9)	0.003
Hematoma, *n* (%)	69 (17.2)	19 (16.0)	0.799
Mortality, *n* (%)	54 (13.5)	1 (0.8)	<0.001

Values are mean ± SD, *n* (%), or median [IQR]. LVO = large vessel occlusion; MeVO = medium vessel occlusion; mTICI = modified thrombolysis in cerebral infarction; NIHSS = National Institutes of Health Stroke Scale; mRS = modified Rankin scale. Tests: χ^2^/Fisher’s exact for categorical variables; Mann–Whitney U or t-test for continuous variables. Bold *p*-values denote significance (<0.05).

**Table 3:** A comparison of the demographical characteristics of patients with primary MeVOs and rescue MeVOs.

Characteristics	MeVOs—Primary (*n* = 119)	MeVOs—Rescue (*n* = 83)	*p*-Value
Demographical characteristics			
Age (years), mean ± SD (range)	70.76 ± 14.18 (14–96)	71.98 ± 13.88 (19–96)	0.545 ^1^
Gender (male), *n* (%)	59 (49.6)	41 (49.4)	0.980 ^2^
Medical history (presence)			
Hypertension, *n* (%)	86 (72.3)	56 (67.5)	0.563 ^3^
Diabetes mellitus, *n* (%)	21 (17.6)	12 (14.5)	0.682 ^3^
Dyslipidemia, *n* (%)	27 (22.7)	23 (27.7)	0.517 ^3^
Atrial fibrillation, *n* (%)	31 (26.1)	25 (30.1)	0.634 ^3^
Smoking, *n* (%)	18 (15.1)	15 (18.1)	0.716 ^3^
Heart valve replacement, *n* (%)	3 (2.5)	0 (0)	0.270 ^4^
A history of stroke, *n* (%)	26 (21.8)	21 (25.3)	0.688 ^3^
Intravenous tPA, *n* (%)	38 (1.9)	28 (33.7)	0.788 ^2^

Data are presented as mean ± SD (ranges: min–max) or median with interquartile range (IQR) for numerical variables; categorical variables are described as number (*n*) and percentage (%). Different small superscript letters in each row show statistically significant differences between groups. ^1^ Independent sample *t*-test. ^2^ Pearson chi-square test. ^3^ Chi-square test with Yates continuity correction. ^4^ Fisher’s exact test. SD: standard deviation; tPA: tissue plasminogen activator; IQR: interquartile range.

**Table 4:** Angiographic and clinical outcomes after mechanical thrombectomy for primary vs. rescue MeVO.

Characteristics	MeVOs—Primary (*n* = 119)	MeVOs—Rescue (*n* = 83)	*p*-Value
Reperfusion, *n* (%)			0.177 ^5^
None	4 (3.4)	8 (9.6)	
mTICI 2b-3	76 (63.9)	48 (57.8)	
mTICI 3	39 (32.8)	27 (32.5)	
Puncture time, median (IQR)	180 (120–260)	210 (140–300)	0.149 ^6^
Puncture-to-recanalization time, median (IQR)	40 (30–55)	38 (29–60)	0.853 ^6^
Admission NIHSS score, median (IQR)	18 (17–19)	19 (17–25)	0.001 ^6^
NIHSS score in the 3rd month, median (IQR)	9.5 (4–12)	13 (8–15)	<0.001 ^6^
mRS score in the 3rd month, *n* (%)			<0.001 ^2^
mRS1	12 (10.1)	3 (3.6)	
mRS2	45 (37.8) ^a^	14 (16.9) ^b^	
mRS3	51 (42.9)	32 (38.6)	
mRS4	10 (8.4) ^a^	16 (19.3) ^b^	
mRS5	1 (0.8) ^a^	18 (21.7) ^b^	
Hematoma, *n* (%)	19 (16)	16 (19.3)	0.672 ^3^
Mortality, *n* (%)	1 (0.8)	18 (21.7)	<0.001 ^3^

Data are presented as mean ± SD (ranges: min–max) or median with interquartile range (IQR) for numerical variables; categorical variables are described as number (*n*) and percentage (%). Different small superscript letters in each row show statistically significant differences between groups. ^2^ Pearson chi-square test. ^3^ Chi-square test with Yates continuity correction. ^5^ Fisher–Freeman–Halton test. ^6^ Mann–Whitney *U* test. SD: standard deviation; tPA: tissue plasminogen activator; mTICI 2b-3: modified thrombolysis in cerebral infarction; NIHSS: National Institutes of Health Stroke Scale; mRS: modified Rankin scale; IQR: interquartile range.

## Data Availability

De-identified data underlying this article are available from the corresponding author upon reasonable request and with appropriate institutional and ethics approvals/data-use agreements.
